# Metastatic Prostate Cancer of Hand

**DOI:** 10.1155/2016/1472932

**Published:** 2016-10-23

**Authors:** Akihito Nagano, Takatoshi Ohno, Koji Oshima, Daichi Ishimaru, Yutaka Nishimoto, Yoshiyuki Ohno, Akihiro Hirakawa, Tatsuhiko Miyazaki, Haruhiko Akiyama

**Affiliations:** ^1^The Department of Orthopaedic Surgery, Gifu University School of Medicine, 1-1 Yanagido, Gifu 501-1193, Japan; ^2^The Department of Orthopaedic Surgery, Japanese Red Cross Gifu Hospital, 3-36 Iwakuracho, Gifu 502-0844, Japan; ^3^The Department of Orthopaedic Surgery, Ibi Kousei Hospital, 2547-4 Miwa, Ibigawa-cho, Gifu 501-0691, Japan; ^4^The Department of Nursing Course, Gifu University School of Medicine, 1-1 Yanagido, Gifu 501-1193, Japan; ^5^The Department of Orthopaedic Surgery, Gifu Municipal Hospital, 7-1 Kashima-cho, Gifu 500-8323, Japan; ^6^The Division of Pathology, Gifu University Hospital, 1-1 Yanagido, Gifu 501-1193, Japan

## Abstract

Soft tissue metastases of prostate cancer to other sites are extremely rare, and, to our best knowledge, there have been no reports of metastasis to soft tissue of the hand. A 63-year-old man was diagnosed with prostatic cancer. During treatment, bone and soft tissue metastases to the right hand, appearing in the first web space, were observed. The tumor was resected, along with both the first and second metacarpal bones. The thumb was reconstructed by pollicization of the remaining index finger, enabling the patient to use the pollicized thumb for activities of daily living. This is the first case report of prostate cancer metastasizing to the soft tissue in hand. After wide resection, pollicization was able to reconstruct a functional hand and thumb.

## 1. Introduction

Prostate cancer has been found to metastasize to the bones, regional lymph nodes, and lungs, with several previous reports describing metastases to other sites. Here, we present the first case of a prostate cancer that metastasized to the soft tissue of the hand.

## 2. Case Presentation

During a routine check-up a 63-year-old man was found to have a high prostate specific antigen (PSA) concentration (7.9 ng/mL). Transrectal fine needle aspiration (FNA) of the prostate provided a definitive diagnosis of poorly differentiated adenocarcinoma (cT3a, Gleason score 8 (4 + 4), 8/8 cores affected). No metastases were detected, and treatment with both the nonsteroidal antiandrogen bicalutamide (Casodex) and goserelin (Zoladex) reduced his PSA level to 0.2 ng/mL within three months. Five years later, however, despite his PSA level remaining low, local extension to the bladder and metastasis to the S1 vertebra were detected. Furthermore, he developed a gradually enlarging painless mass in the first web space of his right hand, adversely affecting his activities of daily living. Therefore he was referred to our department.

**Figure 1:**
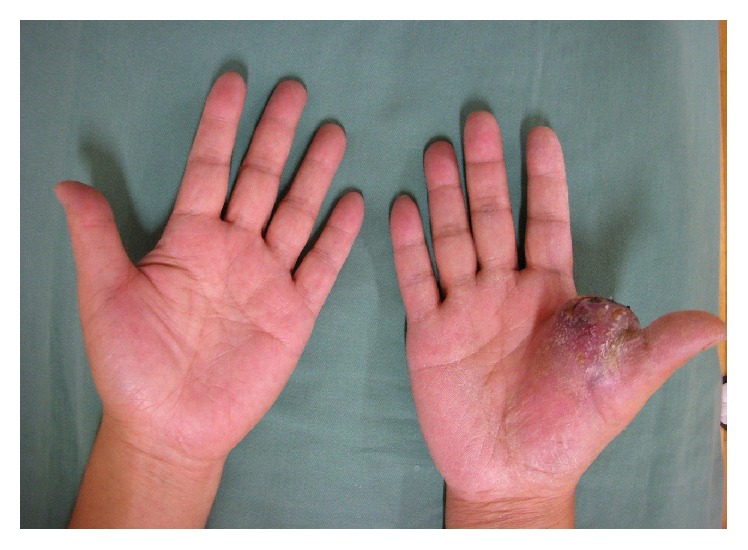
Soft tissue mass in the right hand. The mass was located in the first intercarpal space, with the overlying skin showing discoloration.

Physical examination showed a well-circumscribed, elastic soft mass located between the right thumb and index finger. This mass, which was palpable but not mobile, measured 5 × 4 × 3 cm in size. The skin over the mass was discolored, suggesting that the tumor had invaded the skin. Daily living was impaired due to restricted range of motion (ROM) of the thumb. Although no pain was associated with the mass, the patient experienced sensory disturbance of the right thumb (Figure 1).

Laboratory tests showed elevation of alkaline phosphatase but low PSA level (0.036 ng/mL).

### 2.1. Radiographic Findings

A roentgenogram of the right hand showed enlargement of first metacarpal interspace, indicating noncalcification of the soft tissue mass. The metacarpal bones adjacent to the mass were normal without any bony destruction.

**Figure 2:**
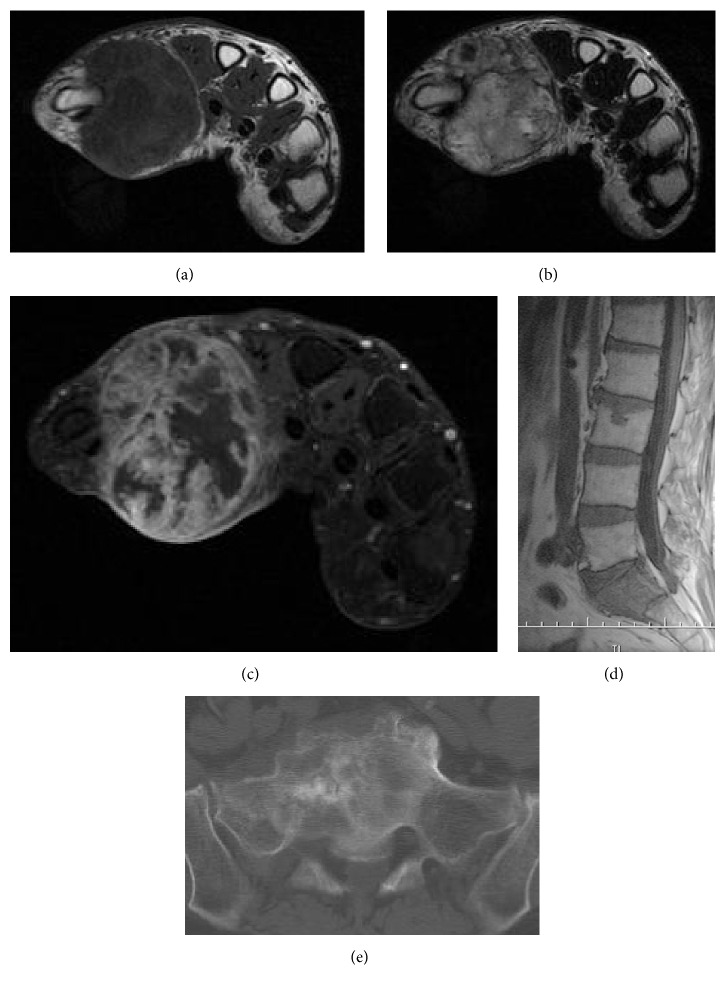
(a) T1 weighted, (b) T2 weighted, and (c) enhanced MRI images of the right hand suggested a malignant neoplasm. (d) MRI and (e) CT of the sacrum showed a lytic and sclerotic lesion, which was regarded as a prostate cancer metastasis.

Magnetic resonance imaging (MRI) of the right hand revealed a well-defined, clearly circumscribed mass, with iso- to low intensity on T1 weighted images and heterogeneously high intensity on T2 weighted images. After intravenous administration of gadolinium-based contrast agent, the mass was well enhanced peripherally, but the central region was poorly enhanced, suggesting necrosis. The mass was adjacent to the first metacarpal bone (Figure 2).

Thallium scintigraphy showed marked accumulation in the right hand but no accumulation in other parts of the body.

Computed tomography (CT) of the entire body revealed a lytic and sclerotic lesion of the sacrum, which was considered metastatic.

Based on these findings, the patient was differentially diagnosed with a primary malignant soft tissue tumor, such as a myxoid liposarcoma or myxofibrosarcoma, or with a metastatic lesion of the adenocarcinoma of the prostate.

### 2.2. Histological Examination

**Figure 3:**
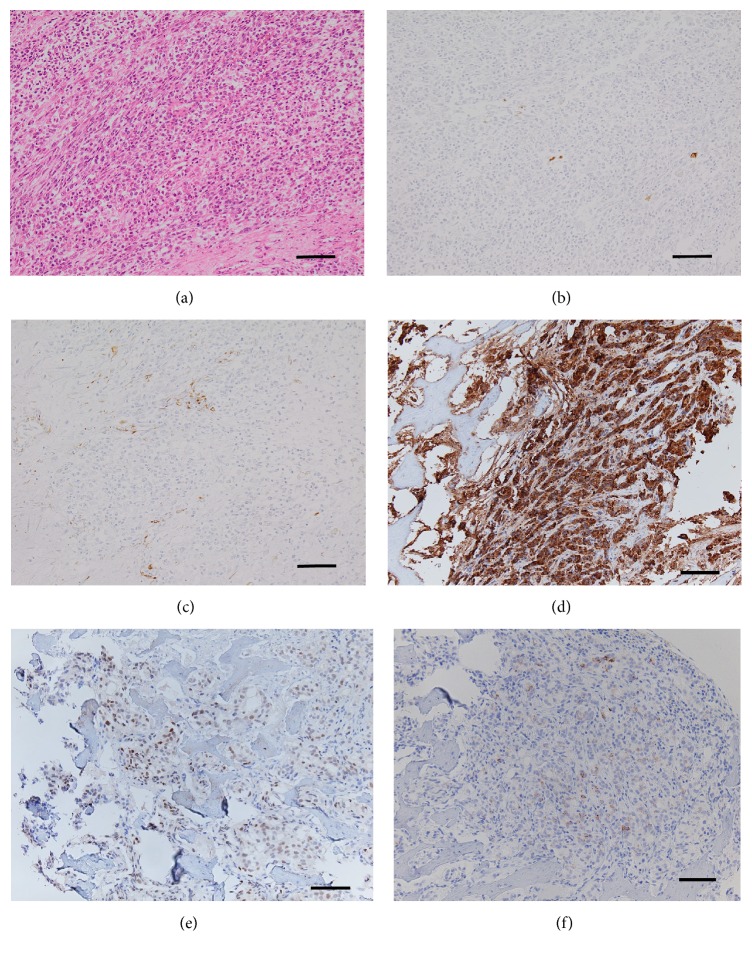
Staining with (a) hematoxylin and eosin (×20), (b) CAM5.2, (c) vimentin, (d) prostate specific acid phosphatase, and (e) androgen receptor showed small-sized but pleomorphic anaplastic tumor cells. (f) Reticulin silver impregnation showed epithelioid-like structures, indicative of carcinoma (bar: 100 *μ*m).

An open biopsy of the mass in the right hand and a CT-guided biopsy of the lesion of the S1 vertebra were obtained to make a definitive diagnosis. Pathological examination of both lesions showed multinodular growth of small-sized but pleomorphic anaplastic tumor cells with numerous mitotic figures. Some tumor cells were plump with eosinophilic cytoplasm. A focal sheet-like arrangement was observed, with no distinct organoid structure. Immunohistochemical evaluation showed that the tumor cells were positive for cytokeratin AE1/AE3, CAM5.2, vimentin, prostate specific acid phosphatase, and androgen receptor and negative for PSA, S-100, CD34, CD68, and smooth muscle actin. Reticulin silver impregnation showed an epithelioid-like structure. These findings indicated that the tumor was an anaplastic carcinoma rather than a mesenchymal malignancy. The patient was therefore diagnosed with metastatic prostate cancer (Figure 3).

### 2.3. Treatment

The treatment of patient with cancer should be performed on a case-by-case basis, depending on the patient's prognosis and functional capabilities especially in patients with multiple metastasis. Generally, palliative treatment, such as radiation therapy, is chosen because of the dismal prognosis of patients with metastasis. In the present case, the prognosis was thought to be relatively better than the other cancer; in fact, the prognosis of prostate cancer with bone metastasis is reported as 3.0–3.5 years [1]. Furthermore, an amputation would bring about unacceptable degree of functional disability, because the affected hand was dominant. Incomplete resection might lead to local recurrence.

After discussion of treatment options with the patient and his family, he agreed to wide resection of the hand tumor and systemic chemotherapy.

**Figure 4:**
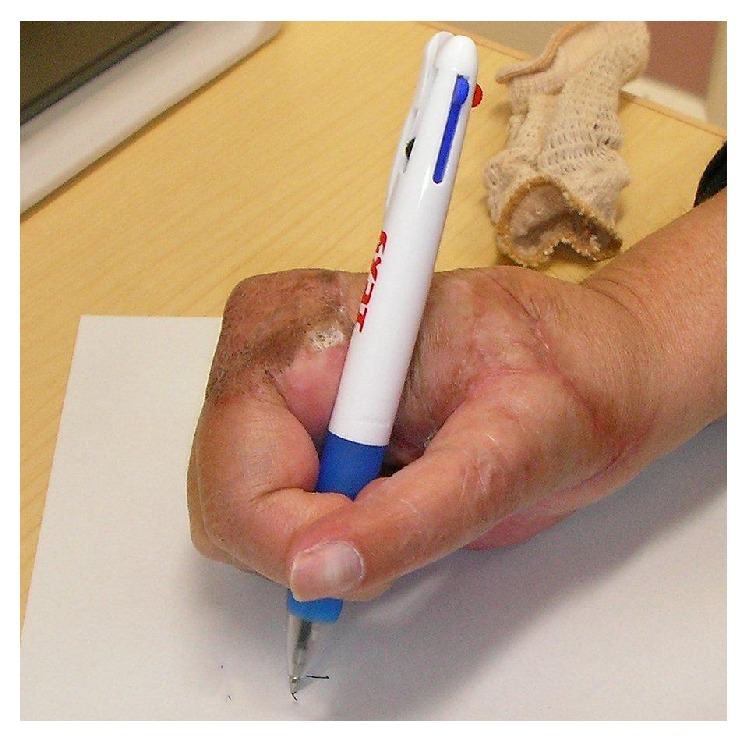
Postoperative appearance of the right hand. The patient used the pollicized thumb for activities of daily living, such as writing.

The tumor, along with the surrounding tissues, was resected* en bloc*. Wide resection of the tumor was accompanied by disarticulation of the carpometacarpal (CMC) joint, osteotomy of the proximal second metacarpal bone, disarticulation of the second metacarpophalangeal (MCP) joint, resection of the tendons and neurovascular bundles of the thumb and index finger, and resection of both the first dorsal interosseous and lumbrical muscles. Concurrently, the thumb was reconstructed by pollicization of the remaining index finger, and the skin defect was covered with a skin graft. The pathological findings of the tumor were the same as the specimen at the time of biopsy, and microscopically free margin (R0 resection) was achieved. After surgery, the patient underwent chemotherapy with docetaxel (DTX) and estramustine. The patient was able to use the pollicized thumb for activities of daily living, such as writing, six months after the operation (Figure 4). Chemotherapy was continued, with no local recurrence in the hand. However, because of multiple metastases to the lungs and spine, the patient died four years after surgery.

## 3. Discussion

The most common locations of prostate cancer metastases are the pelvic lymph nodes and bone, followed by the lungs, bladder, and liver [2]. Soft tissue metastases of prostate cancer to other sites are extremely rare, and, to our knowledge, there have been no reports of metastasis to soft tissue of the hand. Of 47 patients with soft tissue or nonregional lymphatic metastases of prostate carcinoma, 16 had soft tissue metastasis to the lungs, liver, kidneys, bladder, penile glans, subcutaneous tissue of the scalp, thoracic wall, and thigh [3]. Autopsies of 1885 patients with prostate cancer showed metastases in 1367. Sites of metastasis included the lymph nodes, bones, and other visceral organs, but none of these patients had metastases to the hand.

As described previously, a prostate cancer tends to metastasize to the bone. However, acrometastasis, that is, bone metastasis distal to the elbow and the knee, are rare, accounting for approximately 0.1% of all cases with skeletal metastasis. Men were almost twice as likely to acrometastasis as women. The most prevalent primary diagnosis is lung, followed by colon, breast, and genitourinary tract [4, 5]. In the present case, the metastatic tumor was attached to the first metacarpal bone but not invasive, suggesting that metastasis occurred in the soft tissue, not in the bone.

Metastases of well-differentiated prostatic adenocarcinoma show typical pathological features and are not difficult to diagnose. In contrast, metastases of poorly differentiated prostatic adenocarcinoma may present a diagnostic challenge. Immunohistochemical staining, especially for PSA, is useful in assessing the prostatic origin of these less latter tumors. However, higher-grade poorly differentiated adenocarcinoma may lack both pathological features and diagnostic antigenicity, including for PSA [6]. Our patient had an anaplastic carcinoma negative for PSA, making a definitive diagnosis of metastatic prostatic carcinoma quite difficult. The findings that tumor cells were partially positive for both cytokeratin and vimentin and that both the sacral and hand lesions showed similar pathological features and are positive for prostate specific acid phosphatase and androgen receptor strongly suggested that the tumor was metastatic rather than a primary soft tissue sarcoma.

Treatment of patients with advanced prostate cancer must strike a balance between efficacy and acceptable side effects. After assessment of disease risk, patients should be informed about the benefits and side effects of all options [1]. In addition, the presence of bone metastases reduces median survival to about 3.0–3.5 years [1]. The patient in this study was informed of the risks of surgery and prognosis of the disease and chose wide resection in combination with reconstruction of the thumb by pollicization of the index finger, allowing the affected thumb to retain its function. No major complications occurred during or after the operation, and the patient was satisfied with the functional results of the operation.

## 4. Conclusion

We have reported the first case of metastatic prostate adenocarcinoma to the hand. Wide resection of the metastatic tumor and reconstruction of the thumb by pollicization of the index finger resulted in satisfactory clinical and functional outcomes.
